# Novel classification for global gene signature model for predicting severity of systemic sclerosis

**DOI:** 10.1371/journal.pone.0199314

**Published:** 2018-06-20

**Authors:** Zariel I. Johnson, Jacqueline D. Jones, Angana Mukherjee, Dianxu Ren, Carol Feghali-Bostwick, Yvette P. Conley, Cecelia C. Yates

**Affiliations:** 1 Department of Health Promotions and Development, University of Pittsburgh School of Nursing, Pittsburgh, PA, United States of America; 2 Department of Biological & Environmental Sciences, Troy University, Troy, AL, United States of America; 3 Health and Community Systems, University of Pittsburgh School of Nursing, Pittsburgh, PA, United States of America; 4 Department of Rheumatology & Immunology, University of South Carolina, Charleston, SC, United States of America; 5 Department of Human Genetics, University of Pittsburgh, Pittsburgh, PA, United States of America; 6 Department of Pathology, University of Pittsburgh School of Medicine, Pittsburgh, PA, United States of America; University of Bergen, NORWAY

## Abstract

Progression of systemic scleroderma (SSc), a chronic connective tissue disease that causes a fibrotic phenotype, is highly heterogeneous amongst patients and difficult to accurately diagnose. To meet this clinical need, we developed a novel three-layer classification model, which analyses gene expression profiles from SSc skin biopsies to diagnose SSc severity. Two SSc skin biopsy microarray datasets were obtained from Gene Expression Omnibus. The skin scores obtained from the original papers were used to further categorize the data into subgroups of low (<18) and high (≥18) severity. Data was pre-processed for normalization, background correction, centering and scaling. A two-layered cross-validation scheme was employed to objectively evaluate the performance of classification models of unobserved data. Three classification models were used: support vector machine, random forest, and naive Bayes in combination with feature selection methods to improve performance accuracy. For both input datasets, random forest classifier combined with correlation-based feature selection (CFS) method and naive Bayes combined with CFS or support vector machine based recursive feature elimination method yielded the best results. Additionally, we performed a principal component analysis to show that low and high severity groups are readily separable by gene expression signatures. Ultimately, we found that our novel classification prediction model produced global gene signatures that significantly correlated with skin scores. This study represents the first report comparing the performance of various classification prediction models for gene signatures from SSc patients, using current clinical diagnostic factors. In summary, our three-classification model system is a powerful tool for elucidating gene signatures from SSc skin biopsies and can also be used to develop a prognostic gene signature for SSc and other fibrotic disorders.

## Introduction

Systemic Scleroderma (SSc) is a multifaceted disease that exhibits heterogeneity and clinical variability among patients, often complicating diagnosis and decisions regarding treatment. SSc causes skin fibrosis, systemic vascular alterations and collagen accumulation from chronic hardening and tightening of the skin and connective tissues, eventually leading to organ failure and poor prognosis [1]. One level of heterogeneity can be demonstrated by the presence of two SSc disease forms that are defined based on extent of skin involvement: limited cutaneous SSc (lcSSc) and diffuse cutaneous SSc (dcSSc) [2]. Prognosis and risk of internal organ involvement is different for patients in these clinically-defined disease subsets [3–5]. Moreover, genomic studies indicate that variability exists even within each disease forms [6]. Further complexity in SSc stems from the fact that gender [7], race [8], and environmental toxin exposure [9] can predispose individuals to the disease. Thus, the multifactorial nature of SSc pathogenesis can impede accurate measurements of severity and estimation of progression.

Clinical progression of SSc is most often measured by skin thickening using the modified Rodnan skin score (mRSS), which entails a 17-point assessment of skin thickness on various areas of the body, culminating in a 51-point maximum scale of severity. Expression levels of several genes have been associated with skin score [10–12]. However, these investigations did not report the use of a comprehensive analysis comparing various methods available to identify and define a genomic signature that correlates with disease severity. Furthermore, there is evidence that mRSS does not always necessarily correlate with disease trajectory [13]. Thus, the spread of fibrotic behavior to other organs suggests a systemic pathogenic mechanism that requires a more in depth diagnostic analysis to be used in conjunction with skin score. Therefore, the goal of this study was to assess the ability of various classification methods to identify a genetic signature that could be the basis for a diagnostic test for SSc.

In this current study, we constructed classification models with the goal of predicting SSc skin severity based on gene expression profiles and identifying marker gene sets that correlate with high or low severity patients. Ongoing and future studies will focus on the importance of genes identified herein in disease and how they relate to patient-to-patient heterogeneity. Our classification models were capable of readily parsing patients, based on genomic profile alone, into high or low severity groups as defined by mRSS and led to the identification of gene sets associated with disease progression. Thus, our model is a powerful tool not only for diagnosis but also to find previously unknown disease-related genes that warrant further investigation.

## Materials and methods

### Study population

The two datasets used in this study were obtained from the Gene Expression Omnibus (http://www.ncbi.nlm.nih.gov/geo/). Dataset 1 (GEO Accession: GSE9285) [14] contains microarray data from 75 biopsies from 34 individuals, including 28 patients and 6 healthy controls. SSc patients met the criteria of the American College of Rheumatology [15]. Data were generated by Whole Human Genome Oligo Microarray G4112A platform (Agilent Technologies). The skin scores of these samples were documented in the original paper. Microarray data from 21 samples (5 morphea samples, 15 healthy samples, and 1 dSSc sample) was not used because there was no associated skin score information. This left a remaining total of 54 samples, which were included in the analysis. The 54 samples were derived from a total of 24 patients. In several cases, one or more biopsy was taken from a single patient. The skin scores and other patient information related to these samples were documented in the original paper (S1 Table).

Dataset 2 (GEO Accession: GSE47162) [16] contains 59 microarray data of skin biopsies from 59 patients. Data were generated using Illumina HumanHT-12 V4.0 Expression Beadchips. The skin scores and other patient information were extracted from GEO sample record information (S2 Table). One sample without the skin score was excluded from analysis, resulting into a total of 58 samples. For Dataset 1, 25 samples fell into the high severity group and 29 into the low severity group. For Dataset 2, 21 samples fell into the high severity group and 37 into the low severity group.

In all data sets a two-class classification was used to establish classification models that distinguish between "high group" and "low" group ". Skin scores between 18 and 51 were categorized as "high severity", and those less than 18 were categorized as "low severity". An analysis of over 900 patients indicated that a mRSS threshold between 18 and 25 was optimal for distinguishing between patients that would progress or regress [17]. We took a conservative approach and chose the lower limit of this range as our cutoff.

### Statistical analyses

For the Dataset 1, microarrays were visually inspected for defects or technical artifacts, and poor-quality spots were manually excluded in the original study. Spots with fluorescent signals less than two-fold of local background in both Cy3- and Cy5- channel were excluded. Following this pre-processing step, 28,495 probes with at least 80% of their data points were included in downstream analysis. Background correction and quantile normalization were performed using limma package [18]. The data were represented as log2 of the Cy5/Cy3 ratios. Data for each probe were centered by subtracting the expression value from the mean of all expression values across the arrays, and scaled by dividing the centered value by the standard deviation of values across the arrays. For the Dataset 2, quality control was performed by filtering probes using the detection P-value. The detection P-value represents the confidence that a given transcript is expressed above the background level calculated based on negative control probes. We adopted a P-value cutoff of 0.01 as suggested by a quality control manual for this type of microarrays. Probe sets were filtered for a minimum of 20% samples with detection P-value less than 0.01. Quantile normalization was performed across arrays. The data were then log2 transformed, followed by centering and scaling procedures as described for Dataset 1. In total, 9266 features were included for further analysis.

### Cross-validation design

We used a five-fold cross-validation design to compare the performance of the various classification methods. To find the best parameters for the model, we performed another “nested” leave-one-out (LOO) cross-validation on the five original training sets. We identified performance information for each combination of classification method and parameter set and selected the optimal combination to be used for the testing set. This methodology has been described and aims to limit the effects of overfitting the model while objectively evaluating model performance [19].

### Classification

Three classification methods were applied to each of the two datasets: support vector machine (SVM), random forest (RF) and naive Bayes (NB). We used the SVM methods implemented in the e1071 package in R, and selected the RBF (radial basis function) kernel, which has been shown to produce outstanding classification performance in previous studies [19]. We employed the RF implementation in the R package random Forest [20]. There are three important parameters, the values of which need to be determined: mtry (the number of input variable tried at each split), ntree (the number of trees to grow for each forest) and nodesize (the minimum size of the terminal nodes). We considered different parameter configurations for their values of mtry = {0.5,1,2}, ntree = {500,1000,2000} and nodesize = 1, as recommended in a previous study [21]. The best-performing parameters were selected by nested cross-validation, as described in the last section. NB implemented in this study used the klaR package [22] in R. The parameter fL (factor for Laplace correction) was set by default (no correction), and usekernel (logic value to set if a kernel density estimate will be used for density estimation) was optimized by nested cross-validation. Classification model performance was evaluated using three classification metrics: accuracy, sensitivity and specificity. The Matthews Correlation Coefficient (MCC) was also used to quantify the balance between sensitivity and specificity.

### Feature (gene) selection methods

In feature selection method, a small subset of features are identified that, together with the classification methods, are most effective in distinguishing samples belonging to different groups. There are three broad categories of selection methods: filter, wrapper, and embedded methods. Filter methods rank the features or genes regardless of the model. They select the most significant feature or gene based on univariate measure. Wrapper methods evaluate subsets that are optimal with respect to a subset evaluator such as a classifier. Embedded methods incorporate the search algorithm into the classifier. In this study, two filter methods and two wrapper methods were applied. The Chi-Squared method is a filter method that evaluates features or genes individually by measuring their chi-squared statistic with respect to the classes. The correlation-based feature selection method (CFS) method is also a filter method, which measures the correlation between attributes and recognizes those feature subsets in which each feature is highly correlated with the class but uncorrelated with other subset features. The SVM-based recursive feature elimination method (SVM-RFE) is a wrapper method used in microarray data analysis. It eliminates unessential genes and selects better and more compact gene subsets. Random forest-based backward feature elimination method (RFVS) is another wrapper method that constructs RFs in an iterative manner. Upon each iteration, RFVS builds a random forest after discarding genes with the smallest importance values in the last iteration. The returned subset of genes is the one with the smallest out-of-bag error [23].

### Principal component analysis

Principal component analysis (PCA) was performed using function pca() in R (version 3.2.2) package FactoMineR (version 1.31.4). Configuration: scale.unit = TRUE, ncp = 5, ind.sup = NULL, quanti.sup = NULL, quali.sup = NULL, row.w = NULL, col.w = NULL, graph = TRUE, axes = c(1,2) [24].

The component loadings used in PCA are the correlation coefficients between the variables (rows) and factors (columns) and the values for the genes of PC1 and PC2 indicate the "weights" for the genes. The higher the loading value, the higher weight a gene carries in composing the PC. Background correction and quantile normalization were performed using limma package [18]. Data was loaded into MeV as a tab delimited text file of log2-transformed Cy5/Cy3 ratios. For PCA analysis, missing data were first estimated using K-nearest neighbors (KNN) imputation with N = 4.

### Construction of heat maps

Heat maps showing gene expression for the patients by severity group were constructed using normalized and scaled expression levels. Quantile normalization was performed on the raw microarray data, and the normalized expression levels were then Log2 transformed, centered (on 0), and scaled to the same range across genes, so that each gene is evaluated with equal weight.

### Ingenuity Pathway Analysis

Ingenuity Pathway Analysis (Qiagen) (Version 43605062) was used to perform Canonical Pathway Analysis and Upstream Regulator Prediction. Analysis was performed on four lists of microarray probe IDs: identified by CFS classifier in Dataset 1 and differentially expressed, identified by SVM-RFE classifier in Dataset 1 and differentially expressed, identified by CFS classifier in Dataset 2 and differentially expressed, and identified by SVM-RFE classifier in Dataset 2 and differentially expressed. Differential expression was considered statistically significant if the t-test p-value was < 0.05 following Bonferroni correction based on the number of probe IDs identified for each classifier-dataset combination. The Bonferroni correction was applied to limit the false positive discovery rate associated with multiple hypothesis testing. Probe IDs that were successfully mapped by IPA were included for analyses. The direction of expression was positive for probes with higher expression in high severity patients and negative for probes with higher expression in low severity patients. For each dataset, the appropriate reference probe set was based on the microarray used in the original study.

## Results

### Classification and accuracy performances were improved by applying feature selection methods

Several studies, including retrospective cohort analyses and prospective clinical trials, have shown that the severity of skin sclerosis, as assessed by the modified Rodnan skin thickness score (mRSS), is predictive of disease outcome [25]. However, only a very select few have attempted to map the clinical covariates of mRSS to unique gene expression signatures. To further explore this, we used microarray data from two SSc patient studies (Dataset 1 and Dataset 2) from the NCBI GEO Database (Patient data shown in Table 1 and S1 and S2 Tables). We categorized microarray profiles as either low (mRSS 0–17) or high (mRSS 18–51) severity.

**Table 1:** Summary of patient information for microarray biopsy samples used in models.

C	Dataset 1	Dataset 2
**Number of Biopsies**	54	58
**Gender (Male/Female)**	14/40	14/44
**Race (W/AA/H/A)**	44/4/2/4	38/9/10/1
**Age (Years)**	52.3 ± 7.1	52.2 ± 10.7
**Skin Score**	19.5 ± 2.7	14.9 ± 2.7

Means +/- SEM are shown. W: White, AA: African American, H: Hispanic, A: Asian.

We first tested various two-class classification models to determine which could best distinguish between samples designated as low or high severity. The accuracy, sensitivity, specificity, and Mathew’s correlation coefficient (MCC) were determined for each of the classification models (Table 2). MCC values demonstrate the contribution from each of the randomly formed gene sets. An MCC value of 1 indicates a perfect prediction [26]. In the Dataset 1, the accuracy performance classifier RF and feature selection correlation-based selection method CFS showed a MCC value of 0.96 (Table 2). NB combined with either CFS or SVM-RFE showed MCC values of 0.96, and 0.96 respectively (Table 2). In Group 2, RF and CFS had a significant MCC value of 1.00. NB combined with either CFS or SVM-RFE also produced a MCC value of 1.00. Thus, higher MCC values were obtained in cases where either CFS or SVM-RFE selection methods were employed. Therefore, we chose to focus primarily on the results from these two feature selection methods for the remainder of the investigation.

**Table 2:** Performance evaluation of various classifier and feature selection methods.

Dataset	Classifier	Feature Selection	Accuracy	Sensitivity	Specificity	MCC
Dataset 1	SVM	All Features	0.87	0.88	0.87	0.74
		Chi-Squared	0.93	0.96	0.90	0.85
		CFS	0.93	1.00	0.87	0.86
		SVM-RFE	0.96	1.00	0.93	0.93
		RVFS	0.89	0.88	0.90	0.78
	RF	All Features	0.76	0.67	0.83	0.51
		Chi-Squared	0.93	0.92	0.93	0.85
		CFS	0.98	1.00	0.97	0.96
		SVM-RFE	0.94	0.92	0.97	0.89
		RVFS	0.94	0.96	0.93	0.89
	NB	All Features	0.74	0.85	0.73	0.48
		Chi-Squared	0.89	0.83	0.93	0.78
		CFS	0.98	1.00	0.97	0.96
		SVM-RFE	0.98	1.00	0.97	0.96
		RVFS	0.91	0.92	0.90	0.81
Dataset 2	SVM	All Features	0.84	0.62	0.97	0.66
		Chi-Squared	0.91	0.90	0.92	0.82
		CFS	0.95	1.00	0.92	0.90
		SVM-RFE	1.00	1.00	1.00	1.00
		RVFS	0.95	0.95	0.95	0.89
	RF	All Features	0.72	0.29	0.97	0.38
		Chi-Squared	0.91	0.81	0.97	0.81
		CFS	1.00	1.00	1.00	1.00
		SVM-RFE	0.97	0.90	1.00	0.93
		RVFS	0.98	0.95	1.00	0.96
	NB	All Features	0.71	0.43	0.86	0.33
		Chi-Squared	0.90	0.86	0.92	0.78
		CFS	1.00	1.00	1.00	1.00
		SVM-RFE	1.00	1.00	1.00	1.00
		RVFS	0.95	0.95	0.95	0.89

SVM: support vector machine, RF: random forest, NB: naive Bayes, CFS: correlation-based feature selection method, SVM-RFE: SVM-based recursive feature elimination method, RVFS: random forest-based backward feature elimination method, MCC: Mathew’s correlation coefficient.

The CFS selection method identified 84 probe IDs in Dataset 1 and 89 probe IDs in Dataset 2 as being differentially expressed between low and high severity groups. Likewise, the SVM-RFE method identified 450 probe IDs in Dataset 1 and 50 probe IDs in Dataset 2 (Table 3). Specific genes and potential interactions were explored using Ingenuity Pathway Analysis.

**Table 3:** Number of features (microarray probe IDs) selected for each dataset and feature selection method.

Column1	SVM-RFE	Chi-Squared	CFS	RVFS
Dataset 1	450	84	84	23
Dataset 2	50	89	89	9

SVM-RFE: SVM-based recursive feature elimination method, CFS: correlation-based feature selection method, RVFS: random forest-based backward feature elimination method.

### Patient severity classification groups are readily separable by gene expression signatures

**Fig 1:**
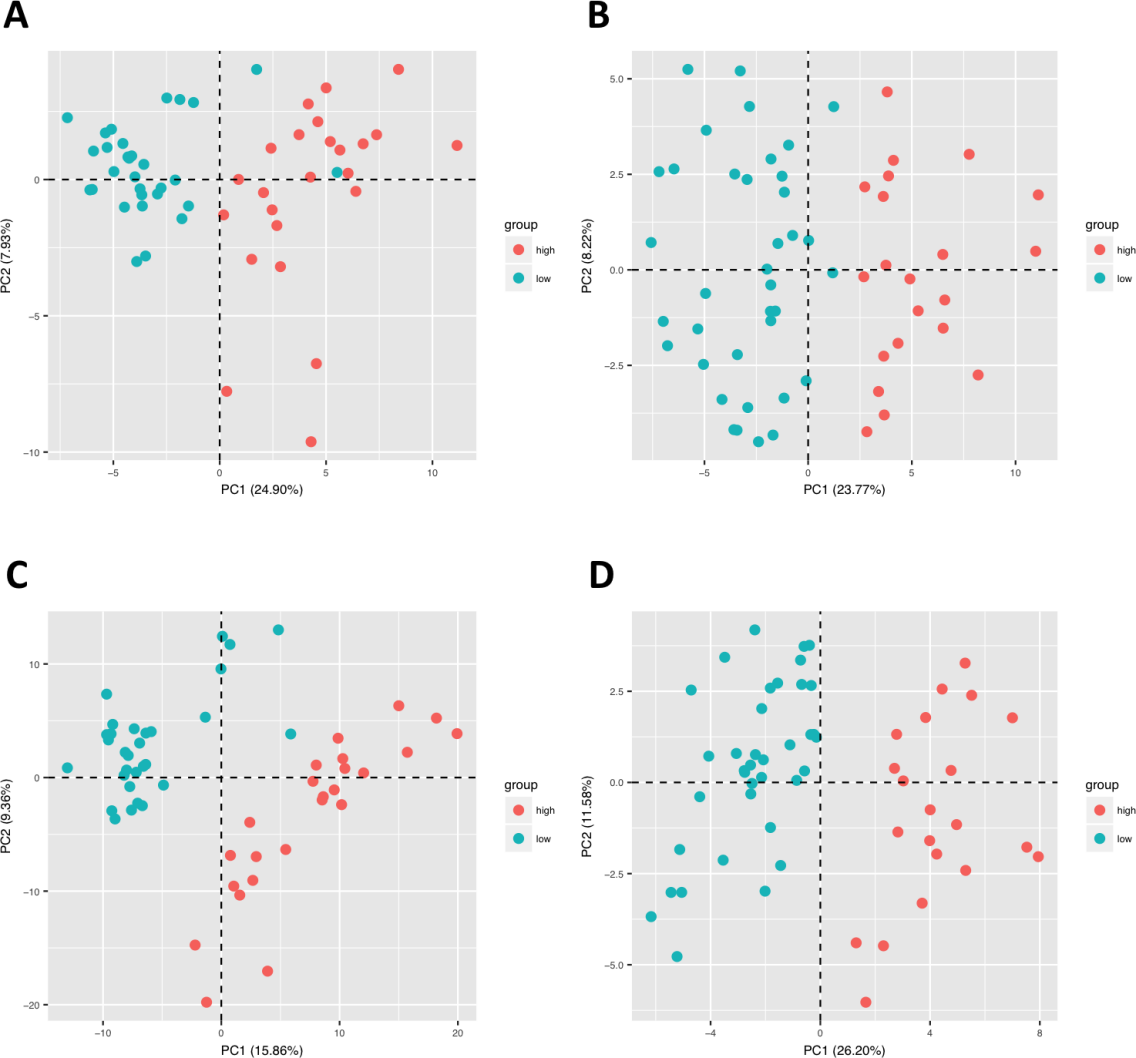
Principal component analyses (PCA) of gene expression separation between low and high severity groups. Results based on CFS feature selection method are shown in A (Dataset 1) and B (Dataset 2). Results based on SVM-RFE feature selection method are shown in C (Dataset 1) and D (Dataset 2).

We performed principal component analysis (PCA) using all targets to pass data cleaning and filtering to visually interpret the variability within and between severity groups. PCA based on all four feature selection methods showed clear separation between severity groups based on gene signature for both Datasets (Fig 1 and S1 Fig). The PCA based on CFS for Dataset 1 showed 24.90% of variance in PC1 and 7.93% of variance in PC2 (Fig 1A). For all feature selection methods and both datasets, PC1 explained much more of the variability than PC2, indicating that the first principal component explained most of the differences between the expression profiles of the high and low severity patients. For the PCA performed on genes from the Chi-squared feature selection method, the microarray probe IDs with the ten highest absolute loading values for PC1 are given in Table 4. Each of these probe IDs was found to be statistically differentially expressed between low and high severity groups, based on Bonferroni-adjusted t-test p-value < 0.05. All The most highly weighted probe IDs for datasets 1 and 2 corresponded to genes for MAGI1 and SOX18, respectively, with positive loading values being associated with the more severe disease phenotype in both cases.

**Table 4:** Microarray probe IDs associated with the top 10 highest absolute value of loading values for principal component 1 based on principal component analysis of genes identified by Chi-squared feature selection.

	Microarray Probe ID	Gene Symbol	Loading Value	Gene Name	Adjusted p-value
**Dataset 1**	A_23_P73297	MAGI1	0.813	membrane associated guanylate kinase, WW and PDZ domain containing 1	1.56E-03
	A_32_P86578	LOC389023	-0.749	uncharacterized LOC389023	1.75E-03
	A_23_P403398	DKFZP586I1420	0.747	uncharacterized protein DKFZp586I1420	1.11E-03
	A_23_P36531	TSPAN8	0.745	tetraspanin 8	1.42E-03
	A_23_P31996	SLC46A2	0.744	solute carrier family 46, member 2	5.80E-04
	A_23_P155441	RFT1	-0.743	RFT1 homolog (S. cerevisiae)	5.19E-03
	A_23_P85140	TCEAL2	0.740	transcription elongation factor A (SII)-like 2	4.19E-03
	A_23_P147647	SGCD	-0.728	sarcoglycan, delta (35kDa dystrophin-associated glycoprotein)	4.40E-03
	A_23_P73220	FGD6	0.727	FYVE, RhoGEF and PH domain containing 6	2.13E-03
	A_23_P92552	PET112	0.726	PET112 homolog (yeast)	1.11E-03
**Dataset 2**	ILMN_1812968	SOX18	0.754	SRY (sex determining region Y)-box 18	8.75E-06
	ILMN_1741688	CPXM2	0.753	carboxypeptidase X (M14 family), member 2	1.10E-05
	ILMN_2402766	AFTPH	-0.750	aftiphilin	2.02E-03
	ILMN_1663618	STAT3	0.742	signal transducer and activator of transcription 3 (acute-phase response factor)	3.23E-03
	ILMN_1676893	ADCY3	0.737	adenylate cyclase 3	5.26E-04
	ILMN_1786197	NR2F1	0.737	nuclear receptor subfamily 2, group F, member 1	9.06E-03
	ILMN_1821397	N/A	-0.737	N/A	6.71E-03
	ILMN_1687840	ABCB7	-0.726	ATP-binding cassette, sub-family B (MDR/TAP), member 7	8.12E-05
	ILMN_1786139	VKORC1	0.726	vitamin K epoxide reductase complex, subunit 1	3.83E-04
	ILMN_1785113	MUT	-0.722	methylmalonyl CoA mutase	2.13E-02

Adjusted p-values are Bonferroni-corrected p-values from T-test statistic comparing normalized levels between low and high severity patient groups.

**Fig 2:**
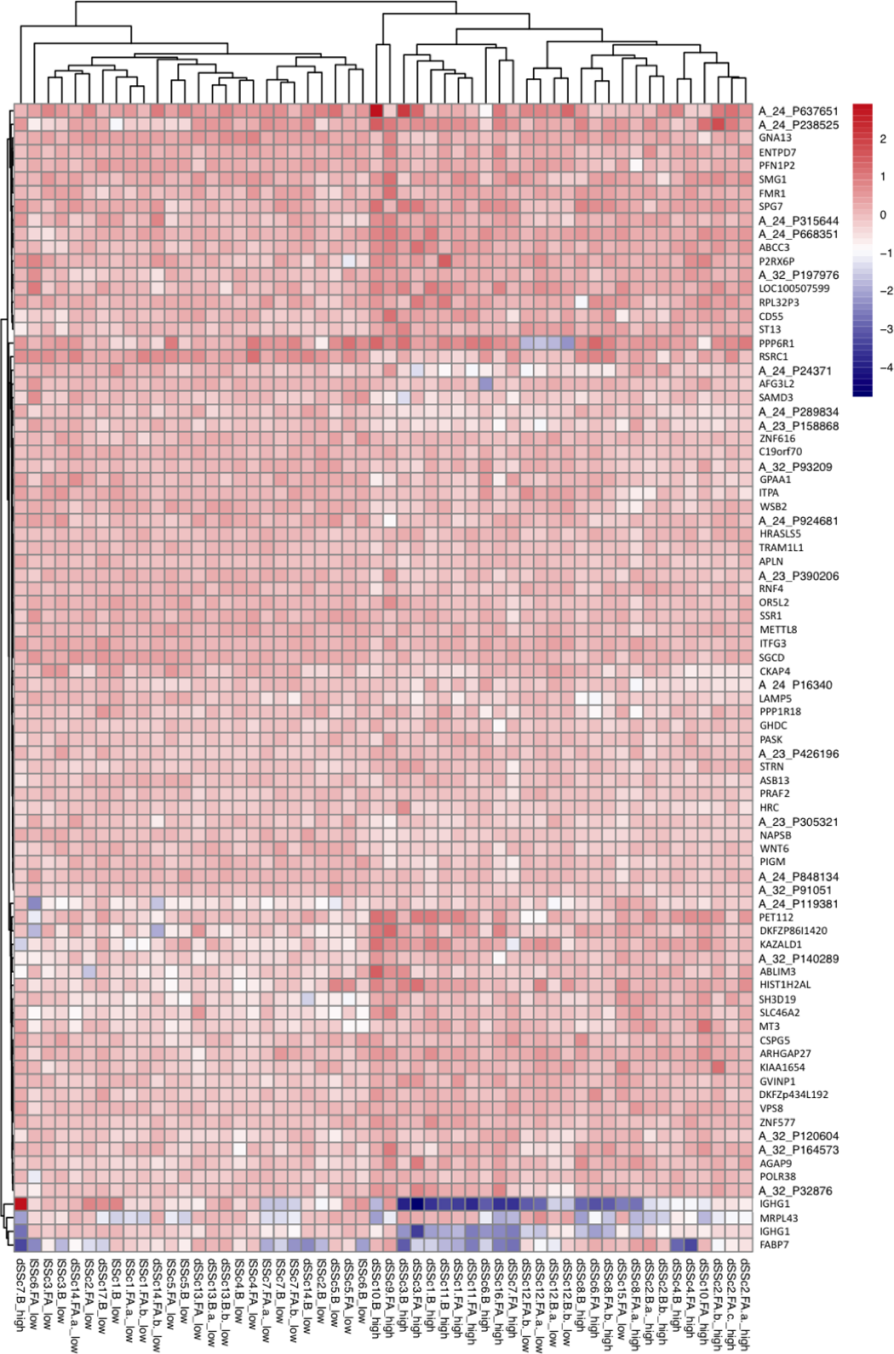
Heat map showing Log2 normalized expression values for patient samples from Dataset 1 for probe IDs identified by CFS feature selection method.

**Fig 3:**
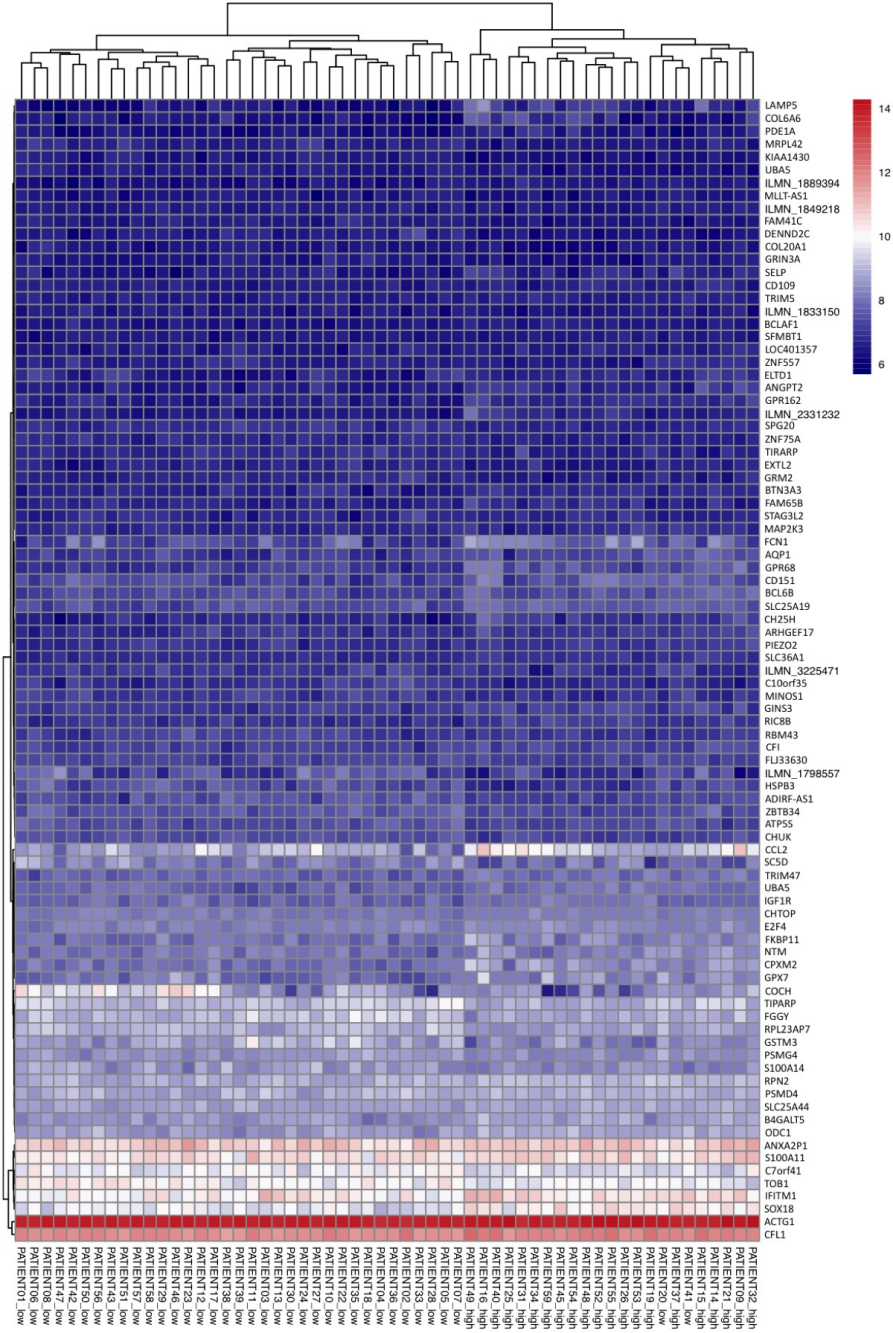
Heat map showing Log2 normalized expression values for patient samples from Dataset 2 for probe IDs identified by CFS feature selection method.

In addition, the Log2 normalized expression values were used to create heat maps for each Dataset. Heat maps were generated to show probe IDs from Dataset 1 (Fig 2) and Dataset 2 (Fig 3) that were identified by CFS feature selection and highlight differences in gene expression profiles between the severity groups. As expected, expression profiles associated with these probe IDs can be separated by severity index when inspected visually. Similar results are shown in heat maps for probe IDs from Dataset 1 (S2 Fig) and 2 (S3 Fig) that were identified by SVM-RFE.

### Pathway enrichment analysis identified a putative signaling network that included genes related to SSc severity

**Fig 4:**
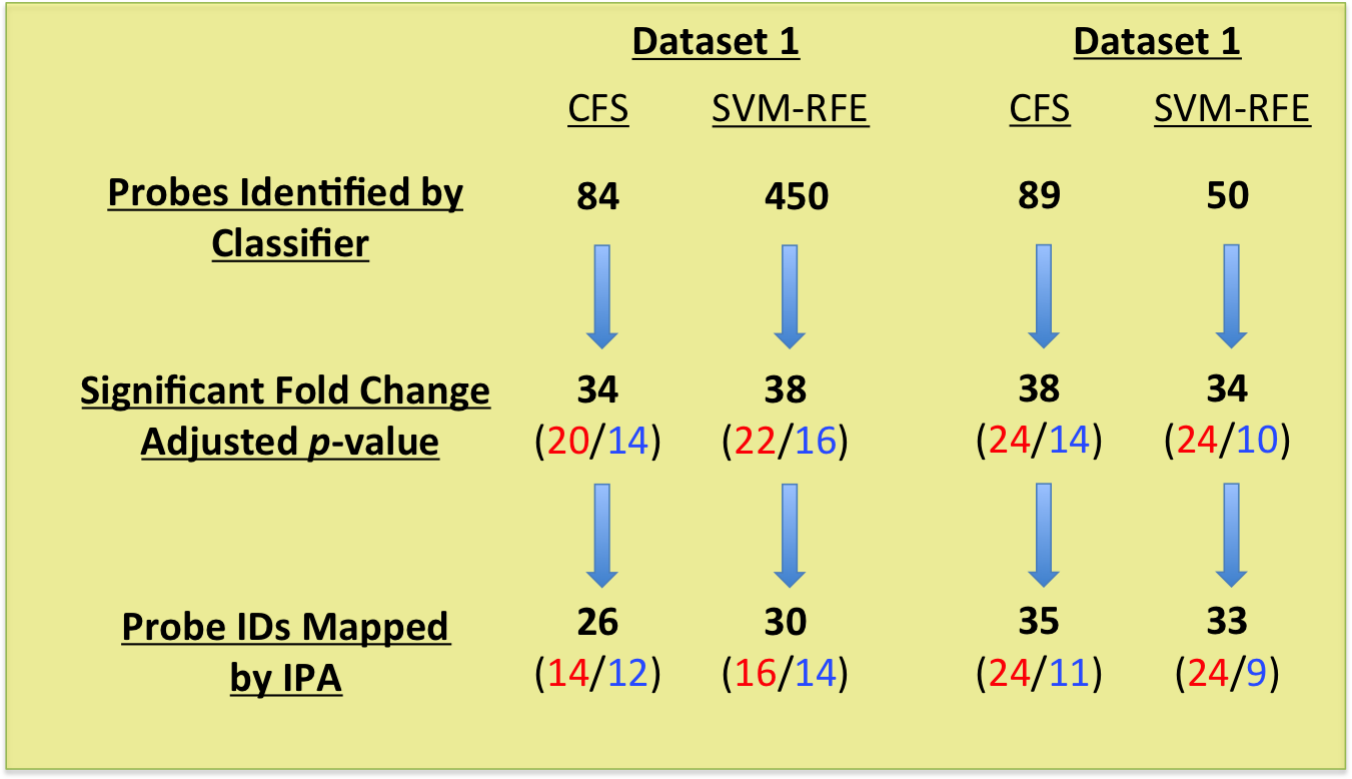
Schematic representation of pipeline for choosing genes that were included in Ingenuity Pathway Analysis. Red font indicates numbers of genes more highly expressed by high severity patients; blue font indicates numbers of genes more highly expressed by low severity patients.

**Fig 5:**
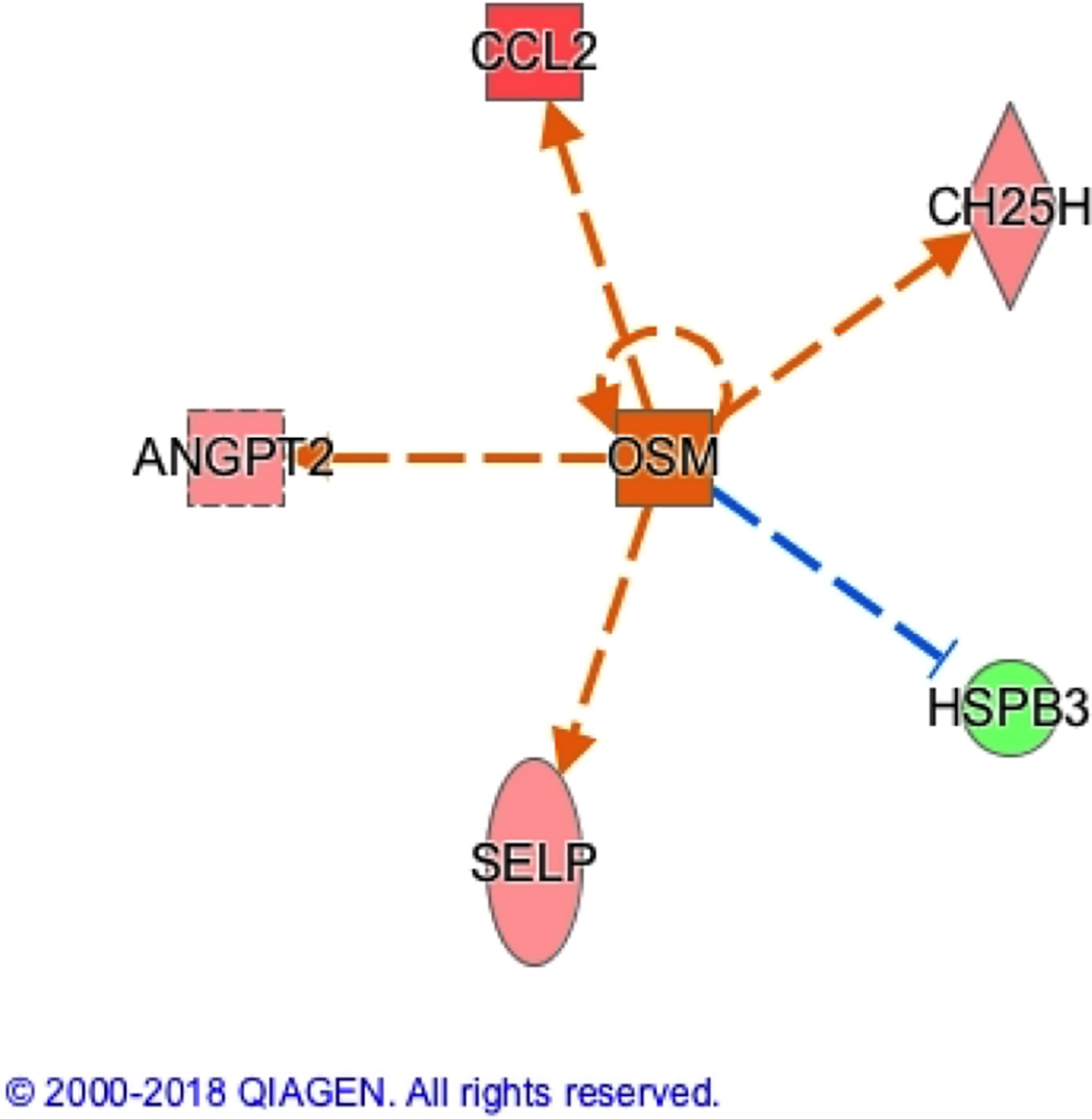
Predicted signaling network between OSM and downstream genes related to SSc severity. Red shading of gene indicates upregulation in dataset compared to low severity patients, green shading downregulation, and intensity of color depicts strength of regulation. Relationships between genes that are predicted based on literature are indicated by lines connecting genes, with red symbolizing predicted upregulation and blue predicted downregulation.

Based on the high performance of our models, we hypothesized that the genes identified by the models and differentially expressed between severity groups may have biological significance. To further explore the relationship between these groups of genes, we used Ingenuity Pathway Analysis (IPA) to analyze the lists of genes that were both identified by the classifier and that showed differential expression between severity groups (Bonferroni-corrected t-test p-value < 0.05). Fig 4 depicts the numbers of Probe IDs that were identified by the classifier, were statistically significant, and mapped to Gene IDs for each classifier and dataset combination. We first investigated whether our gene lists were overrepresented in known Canonical Pathways. The top three Canonical Pathways (ranked by Benjamini-Hochberg p-value) are shown in S3 Table. Our analysis failed to find any pathways that showed statistically meaningful overrepresentation of our input genes. We then used the Upstream Regulator Analysis in IPA to see whether a common upstream regulator could explain the differences that we saw in gene expression between low and high severity patient samples. The upstream regulator that showed the most predictive value, based on a combination of activation Z-score of 2.159 and p-value of overlap of 2.25E-03 was oncostatin M (OSM). Activation of OSM was predicted to regulate five of the genes with significant expression differences identified in Dataset 2 using the CFS classifier when comparing low and high severity patients (Table 5 and Fig 5).

**Table 5:** Results of IPA-based upstream regulator analysis showing potential role for OSM in regulating genes identified by CFS-based classification of Dataset 2 and differentially expressed between low and high severity patients.

Probe ID	Genes in Dataset	Prediction of OSM Activation (based on measurement direction)	High vs. Low Severity Regulation	Evidence from Literature
ILMN_1715417	SELP	Activated	Upregulated	Upregulated by OSM1
ILMN_1720710	HSPB3	Activated	Downregulated	Downregulated by OSM1
ILMN_1741021	CH25H	Activated	Upregulated	Upregulated by OSM1
ILMN_1720048	CCL2	Activated	Upregulated	Upregulated by OSM1
ILMN_3250067	ANGPT2	Activated	Upregulated	Upregulated by OSM1

## Discussion

This study is one of the first to describe an independently validated classification prediction model for gene signatures in SSc using current clinical diagnostic factors. We used applied three well-established classification methods, SVM, RF, and NB, coupled with four different feature selections, to clinically derived microarray data and compared the relative efficiency of the models. Interestingly, RF and CFS showed high MCC values of 0.96 and 1.00 for both of the independent datasets that we used, suggesting an efficient diagnostic two-layered system for SSc. This two-layered model system is capable of reducing the dimensionality of large independent data to find a more useful subset for further exploration. Moreover, due to the heterogeneity of patients with SSc, a prediction model that can deduce disease severity in an unsupervised manner is essential. To get an idea of how the gene lists generated by our classifiers compared to conventionally-derived differentially expressed genes, we also performed a formal differential gene expression analysis comparing low and high severity patients from each dataset (data not shown), which identified many probe IDs that were statistically significant between groups. While some genes identified by the feature selection methods overlapped with this list of conventionally derived differentially expressed genes, in each case our classifiers also identified several genes that were not found with the conventional differential gene analysis. This highlights the value of our classifiers in finding genes that can be useful for classification purposes but may not be identified by conventional methods.

Further validating the efficiency of the two-step model system, principal component analysis showed a clear separation of patients with high and low skin scores when subjected to the model. This clustering of datasets further substantiates the efficiency of the model system to reduce complexity and distinguish between high and low severity gene profiles. After performing the PCA, we examined which microarray probe IDs were associated with the highest loading values to gain a sense of which were the most important in separating the severity groups. The most highly weighted probe IDs were associated with a broad array of biological processes. In Dataset 1, Tetraspanin 8 showed one of the highest loading levels. Tetraspanins have previously been shown to alter fibrosis through the regulation of epithelial cell-basement membrane interaction [27]. For Dataset 2, we found that the gene Signal Transducer and Activator of Transcription 3 (STAT3) ranked was one of those with the highest loading value. STAT3 has been extensively associated with dermal fibrosis and is critical in the pro-fibrotic effects of TGF-β signaling [28,29]. Our analysis suggests that the other highly weighted genes are key in distinguishing low and high severity patients and should be carefully considered for further investigation. Based on the results of the CFS feature selection method, heat maps showing Log2 normalized expression for both datasets showed a clear distinction between patients with high and low skin scores, signifying the presence of two separately clustered gene profiles.

Our model assigned patients into high and low severity groups using skin score, the standard diagnostic measurement for SSc. This bolsters the efficiency and effectiveness of our model system to determine outcome, which should help decrease the lack of specificity that currently hampers clinical treatment. The analytical pipeline reported herein highlights the potential applicability of an unsupervised, tiered correlation method, whereby disease severity can be classified based upon genetic signature alone. Furthermore, the novel gene sets that have been identified as correlating with disease severity may be further investigated to provide insight into the molecular mechanisms underlying a clinical trait of SSc, a complex fibrotic disease. Our methodology can be used to stratify patients to assess response to therapy and to guide appropriate recruitment to clinical trials.

To evaluate the functional relevance of these clusters in the context of disease, we used various tools within Ingenuity Pathway Analysis to explore interactions between genes that were both identified by our classification methods and showed differential expression between low and high severity groups. Our analysis did not return any pathways that were significantly enriched by the genes in our lists, showing that the genes identified here to do not belong to known biological pathways. This result indicates that our classification methods are capable of identifying genes that, when taken together, can accurately be used to predict disease severity although they may not participate in the same pathways *in vivo*. Additionally, it is possible that these genes may interact in pathways that are not yet known. However, our analysis of potential upstream regulators did identify one small network, controlled by the cytokine oncostatin-m (OSM), that could drive relatively high expression of P-selectin (SELP), cholesterol 24-hydroxylase (CH25H), c-c motif chemokine ligand 2 (CCL2), and angiopoietin 2 (ANGPT2) and low expression of heat shock protein family B member 3 (HSPB3) in higher severity SSc patients. Evidence in the literature shows that OSM is capable of regulating these genes with directionality suggesting that OSM positively regulates the genes associated with the more severe skin scores [30–32]. Studies have shown that levels of OSM, an IL-6 family cytokine, are associated with SSc and OSM can modulate production of several extracellular matrix components important in fibrosis [33–36]. Relevant to our study, serum levels of soluble angiopoietin-1, p-selectin, and CCL2 correlate with increasing severity of SSc and worsening clinical features [37–40]. Therefore, our data suggest that OSM may influence the expression of additional genes associated with severe disease.

We observed some differences in the probe IDs identified by our classification methods and in subsequent downstream analyses. For the two datasets, the CFS method identified similar number of probe IDs that were used for classification (84 and 85 for Datasets 1 and 2, respectively). On the other hand, the SVM-RFE method identified 450 probe IDs for Dataset 1 and 50 for Dataset 2. We believe that these differences stem from the fact that CFS and SVM-RFE use intrinsically different methods for feature selection. The CFS method is a filter method that gives priority to genes whose expression is highly correlated with a class. SFM-RFE is a wrapper method that discards genes that have only a small impact on classification. Interestingly, when only the genes that have significantly different expression levels between severity groups are considered, the numbers of genes identified by the classifiers are more analogous. This indicates that the majority of the 450 probe IDs identified when the SVM-RFE method was applied to Dataset 2 were useful for classification, despite not being considered differentially expressed between groups.

Our novel three-classification model system of SVM, RF, and NB is an efficient and powerful tool for developing gene signatures from SSc skin biopsies and can also be used to develop a prognostic gene signature for SSc. Our models achieved high accuracy, specificity, and sensitivity, demonstrating their potential use as diagnostic indicators. Furthermore, our model system can be used to model other fibrotic disorders by substituting different phenotypes in the training cohort. Current ongoing and future studies using alternative methodologies will investigate specific genes identified by the classifiers to determine how they may relate to disease pathogenesis and progression.

## Supporting information

S1 Table Patient information for samples used from Dataset 1. B: Back biopsy, FA: Forearm biopsy, F: Female, M: Male, W: White, A: Asian, AA: African American, H: Hispanic. Shading indicates individual patients.
(DOCX)

S2 Table Patient information for samples used from Dataset 2. F: Female, M: Male, W: White, A: Asian, AA: African American, H: Hispanic.
(DOCX)

S3 Table Canonical pathways identified by Ingenuity Pathway Analysis. Probe IDs included in analysis were both identified by the classification method and significantly differentially expressed between low and high severity groups (Bonferroni-corrected t-test p-value < 0.05). CFS: correlation-based feature selection method, SVM-RFE: SVM-based recursive feature elimination method. Ratio: number of genes from input list that occur in pathway. B-H p-value: Benjamini-Hochberg adjusted p-value of right-tailed Fisher’s Exact Test.
(DOCX)

S1 Fig Principal component analysis (PCA) of gene expression separation between low and high severity groups. Results based on Chi-squared feature selection method are shown in A (Dataset 1) and B (Dataset 2). Results based on RVFS feature selection method are shown in C (Dataset 1) and D (Dataset 2).
(TIF)

S2 Fig Heat map showing Log2 normalized expression values for patient samples from Dataset 1 for probe IDs identified by SVM-RFE feature selection method. (TIF)

S3 Fig Heat map showing Log2 normalized expression values for patient samples from Dataset 2 for probe IDs identified by SVM-RFE feature selection method. (TIF)
