# Supplementary material for: Novel classification for global gene signature model for predicting severity of systemic sclerosis
Source: PLoS One. 2018 Jun 20;13(6):e0199314. doi: 10.1371/journal.pone.0199314 (PMC6010260; doi:10.1371/journal.pone.0199314)
Supplement: S1 Table — B: Back biopsy, FA: Forearm biopsy, F: Female, M: Male, W: White, A: Asian, AA: African American, H: Hispanic. Shading indicates individual patients. (DOCX) [file pone.0199314.s001.docx]

**S1 Table. Patient information for samples used from Dataset 1.** B: Back biopsy, FA: Forearm biopsy, F: Female, M: Male, W: White, A: Asian, AA: African American, H: Hispanic. Shading indicates individual patients.

| **Patient** | **GEO accession ID** | **Age** | **Gender** | **Race** | **Disease Type** | **Skin Score** | **Severity Group** |
| --- | --- | --- | --- | --- | --- | --- | --- |
| dSSc1-B | GSM236379 | 41 | F | W | dSSc | 28 | high |
| dSSc1-FA | GSM236380 | 41 | F | W | dSSc | 28 | high |
| dSSc2-B(a) | GSM236381 | 49 | M | W | dSSc | 26 | high |
| dSSc2-B(b) | GSM236382 | 49 | M | W | dSSc | 26 | high |
| dSSc2-FA(a) | GSM236383 | 49 | M | W | dSSc | 26 | high |
| dSSc2-FA(b) | GSM236384 | 49 | M | W | dSSc | 26 | high |
| dSSc2-FA(c) | GSM236385 | 49 | M | W | dSSc | 26 | high |
| dSSc3-B | GSM236386 | 33 | F | H | dSSc | 35 | high |
| dSSc3-FA | GSM236387 | 33 | F | H | dSSc | 35 | high |
| dSSc4-B | GSM236388 | 47 | F | W | dSSc | 35 | high |
| dSSc4-FA | GSM236389 | 47 | F | W | dSSc | 35 | high |
| dSSc5-B | GSM236390 | 52 | F | W | dSSc | 10 | low |
| dSSc5-FA | GSM236391 | 52 | F | W | dSSc | 10 | low |
| dSSc6-B | GSM236392 | 63 | F | W | dSSc | 26 | high |
| dSSc6-FA | GSM236393 | 63 | F | W | dSSc | 26 | high |
| dSSc7-B | GSM236394 | 43 | F | W | dSSc | 23 | high |
| dSSc7-FA | GSM236395 | 43 | F | W | dSSc | 23 | high |
| dSSc8-B | GSM236396 | 58 | M | A | dSSc | 43 | high |
| dSSc8-FA(a) | GSM236397 | 58 | M | A | dSSc | 43 | high |
| dSSc8-FA(b) | GSM236398 | 58 | M | A | dSSc | 43 | high |
| dSSc9-FA | GSM236450 | 58 | F | W | dSSc | 21 | high |
| dSSc10-B | GSM236399 | 35 | F | AA | dSSc | 35 | high |
| dSSc10-FA | GSM236400 | 35 | F | AA | dSSc | 35 | high |
| dSSc11-B | GSM236401 | 47 | F | W | dSSc | 30 | high |
| dSSc11-FA | GSM236402 | 47 | F | W | dSSc | 30 | high |
| dSSc12-B(a) | GSM236403 | 58 | M | W | dSSc | 15 | low |
| dSSc12-B(b) | GSM236404 | 58 | M | W | dSSc | 15 | low |
| dSSc12-FA(a) | GSM236405 | 58 | M | W | dSSc | 15 | low |
| dSSc12-FA(b) | GSM236406 | 58 | M | W | dSSc | 15 | low |
| dSSc13-B(a) | GSM236407 | 47 | F | W | dSSc | 15 | low |
| dSSc13-B(b) | GSM236408 | 47 | F | W | dSSc | 15 | low |
| dSSc13-FA | GSM236409 | 47 | F | W | dSSc | 15 | low |
| dSSc14-B | GSM236410 | 49 | F | W | dSSc | 15 | low |
| dSSc14-FA(b) | GSM236411 | 49 | F | W | dSSc | 15 | low |
| dSSc14-FA(a) | GSM236412 | 49 | F | W | dSSc | 15 | low |
| dSSc15-FA | GSM236451 | 65 | F | W | dSSc | 18 | high |
| dSSc16-FA | GSM236452 | 40 | F | A | dSSc | 20 | high |
| dSSc17-B | GSM236453 | 56 | F | W | dSSc | 15 | low |
| lSSc1-B | GSM236413 | 67 | F | W | lSSc/CREST | 8 | low |
| lSSc1-FA(a) | GSM236414 | 67 | F | W | lSSc/CREST | 8 | low |
| lSSc1-FA(b) | GSM236415 | 67 | F | W | lSSc/CREST | 8 | low |
| lSSc2-B | GSM236416 | 57 | M | W | lSSc/CREST | 8 | low |
| lSSc2-FA | GSM236417 | 57 | M | W | lSSc/CREST | 8 | low |
| lSSc3-B | GSM236418 | 35 | F | W | lSSc/CREST | 6 | low |
| lSSc3-FA | GSM236419 | 35 | F | W | lSSc/CREST | 6 | low |
| lSSc4-B | GSM236420 | 63 | F | W | lSSc/CREST | 8 | low |
| lSSc4-FA | GSM236421 | 63 | F | W | lSSc/CREST | 8 | low |
| lSSc5-B | GSM236422 | 60 | F | AA | lSSc/CREST | 9 | low |
| lSSc5-FA | GSM236423 | 60 | F | AA | lSSc/CREST | 9 | low |
| lSSc6-B | GSM236424 | 55 | F | W | lSSc/CREST | 9 | low |
| lSSc6-FA | GSM236425 | 55 | F | W | lSSc/CREST | 9 | low |
| lSSc7-B | GSM236426 | 67 | F | W | lSSc/CREST | 8 | low |
| lSSc7-FA(a) | GSM236427 | 67 | F | W | lSSc/CREST | 8 | low |
| lSSc7-FA(b) | GSM236428 | 67 | F | W | lSSc/CREST | 8 | low |
