# Supplementary material for: Novel classification for global gene signature model for predicting severity of systemic sclerosis
Source: PLoS One. 2018 Jun 20;13(6):e0199314. doi: 10.1371/journal.pone.0199314 (PMC6010260; doi:10.1371/journal.pone.0199314)
Supplement: S2 Table — F: Female, M: Male, W: White, A: Asian, AA: African American, H: Hispanic. (DOCX) [file pone.0199314.s002.docx]

**S2 Table. Patient information for samples used from Dataset 2.** F: Female, M: Male, W: White, A: Asian, AA: African American, H: Hispanic.

| **GEO accession ID** | **Age** | **Gender** | **Race** | **Skin Score** | **Severity Group** |
| --- | --- | --- | --- | --- | --- |
| GSM1145868 | 22 | F | AA | 4 | low |
| GSM1145869 | 24 | F | AA | 6 | low |
| GSM1145870 | 24 | F | H | 7 | low |
| GSM1145871 | 31 | F | AA | 11 | low |
| GSM1145872 | 35 | F | H | 4 | low |
| GSM1145873 | 38 | F | W | 4 | low |
| GSM1145874 | 39 | F | W | 3 | low |
| GSM1145875 | 43 | F | W | 9 | low |
| GSM1145876 | 45 | F | AA | 33 | high |
| GSM1145877 | 49 | F | W | 14 | low |
| GSM1145878 | 49 | F | AA | 16 | low |
| GSM1145879 | 50 | F | H | 5 | low |
| GSM1145880 | 53 | F | AA | 9 | low |
| GSM1145881 | 54 | F | W | 31 | high |
| GSM1145882 | 55 | F | W | 27 | high |
| GSM1145883 | 58 | F | AA | 39 | high |
| GSM1145884 | 58 | F | H | 15 | low |
| GSM1145885 | 58 | F | A | 9 | low |
| GSM1145886 | 60 | F | W | 20 | high |
| GSM1145887 | 64 | F | W | 18 | low |
| GSM1145888 | 65 | F | W | 32 | high |
| GSM1145889 | 69 | F | W | 2 | low |
| GSM1145890 | 74 | F | W | 2 | low |
| GSM1145891 | 82 | F | W | 2 | low |
| GSM1145892 | 29 | M | AA | 29 | high |
| GSM1145893 | 46 | M | W | 26 | high |
| GSM1145894 | 46 | M | W | 8 | low |
| GSM1145895 | 50 | M | W | 5 | low |
| GSM1145896 | 63 | M | W | 12 | low |
| GSM1145897 | 50 | M | H | 18 | low |
| GSM1145898 | 59 | F | W | 20 | high |
| GSM1145899 | 46 | F | W | 19 | high |
| GSM1145900 | 62 | F | W | 8 | low |
| GSM1145901 | 30 | F | W | 27 | high |
| GSM1145902 | 54 | F | W | 6 | low |
| GSM1145903 | 58 | F | W | 2 | low |
| GSM1145904 | 25 | M | W | 19 | high |
| GSM1145905 | 50 | F | W | 17 | low |
| GSM1145906 | 62 | F | W | 6 | low |
| GSM1145907 | 65 | M | H | 32 | high |
| GSM1145908 | NA | M | W | 13 | low |
| GSM1145909 | 63 | F | W | 4 | low |
| GSM1145910 | 64 | F | W | 18 | low |
| GSM1145912 | 63 | F | W | 32 | high |
| GSM1145913 | 66 | F | W | 3 | low |
| GSM1145914 | 61 | F | H | 10 | low |
| GSM1145915 | 52 | F | W | 23 | high |
| GSM1145916 | 52 | M | W | 34 | high |
| GSM1145917 | 57 | F | W | 14 | low |
| GSM1145918 | 66 | M | W | 11 | low |
| GSM1145919 | 35 | F | H | 20 | high |
| GSM1145920 | 53 | F | W | 24 | high |
| GSM1145921 | 49 | F | W | 24 | high |
| GSM1145922 | 44 | M | H | 26 | high |
| GSM1145923 | 52 | M | W | 6 | low |
| GSM1145924 | 71 | M | W | 4 | low |
| GSM1145925 | 56 | F | H | 2 | low |
| GSM1145926 | 80 | F | AA | 23 | high |
