# Supplementary material for: Novel classification for global gene signature model for predicting severity of systemic sclerosis
Source: PLoS One. 2018 Jun 20;13(6):e0199314. doi: 10.1371/journal.pone.0199314 (PMC6010260; doi:10.1371/journal.pone.0199314)
Supplement: S3 Table — Probe IDs included in analysis were both identified by the classification method and significantly differentially expressed between low and high severity groups (Bonferroni-corrected t-test p-value < 0.05). CFS: correlation-based feature selection method, SVM-RFE: SVM-based recursive feature elimination method. Ratio: number of genes from input list that occur in pathway. B-H p-value: Benjamini-Hochberg adjusted p-value of right-tailed Fisher’s Exact Test. (DOCX) [file pone.0199314.s003.docx]

**S3 Table. Canonical pathways identified by Ingenuity Pathway Analysis.** Probe IDs included in analysis were both identified by the classification method and significantly differentially expressed between low and high severity groups (Bonferroni-corrected t-test p-value < 0.05). CFS: correlation-based feature selection method, SVM-RFE: SVM-based recursive feature elimination method. Ratio: number of genes from input list that occur in pathway. B-H p-value: Benjamini-Hochberg adjusted p-value of right-tailed Fisher’s Exact Test.

|  | **Canonical Pathway** | **Ratio** | **B-H p-value** |
| --- | --- | --- | --- |
| **Dataset 1 - CFS** | L-glutamine Biosynthesis II (tRNA-dependent) | 50% (1/2) | 0.1315 |
|  | Phospholipase C Signaling | 0.9% (2/224) | 0.3133 |
|  | Hematopoiesis from Pluripotent Stem Cells | 2.9% (1/34) | 0.3133 |
|  |  |  |  |
| **Dataset 1 - SVM-RFE** | L-glutamine Biosynthesis II (tRNA-dependent) | 50% (1/2) | 0.1312 |
|  | Axonal Guidance Signaling | 0.7% (3/444) | 0.3083 |
|  | Hematopoiesis from Pluripotent Stem Cells | 2.9% (1/34) | 0.3083 |
|  |  |  |  |
| **Dataset 2 - CFS** | Chemokine Signaling | 2.9% (2/68) | 0.2438 |
|  | Superoxide Radicals Degradation | 12.5% (1/8) | 0.2438 |
|  | Atherosclerosis Signaling | 1.6% (1/123) | 0.2438 |
|  |  |  |  |
| **Dataset 2 - SFM-RFE** | Molecular Mechanisms of Cancer | 1.0% (4/388) | 0.2275 |
|  | Breast Cancer Regulation by Stathmin1 | 1.5% (3/204) | 0.2275 |
|  | Leptin Signaling in Obesity | 2.4% (2/85) | 0.2410 |
