# Supplementary figures and images for: Novel classification for global gene signature model for predicting severity of systemic sclerosis
Source: PLoS One. 2018 Jun 20;13(6):e0199314. doi: 10.1371/journal.pone.0199314 (PMC6010260; doi:10.1371/journal.pone.0199314)

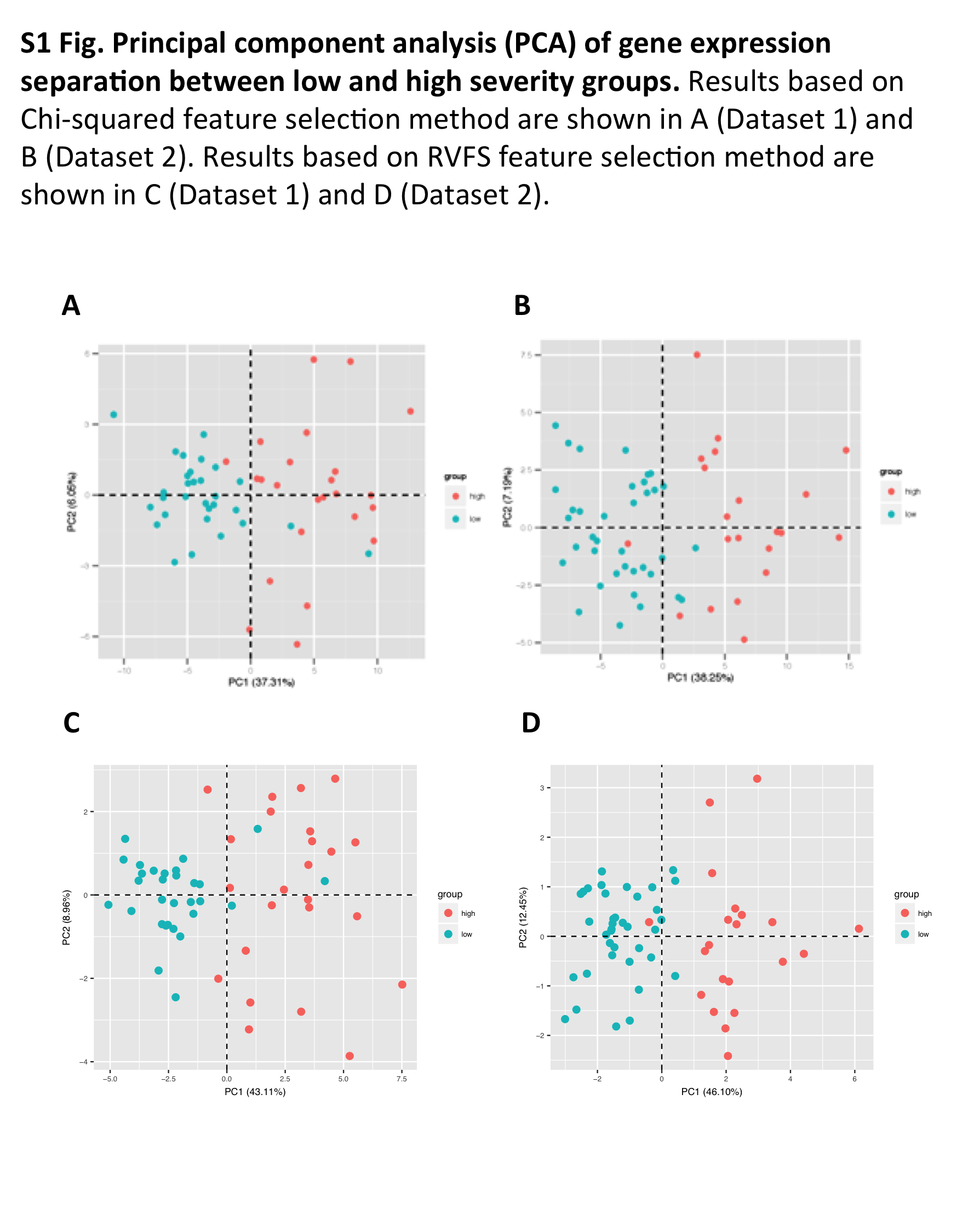

Supplement: S1 Fig — Results based on Chi-squared feature selection method are shown in A (Dataset 1) and B (Dataset 2). Results based on RVFS feature selection method are shown in C (Dataset 1) and D (Dataset 2). (TIF) [file pone.0199314.s004.tif]

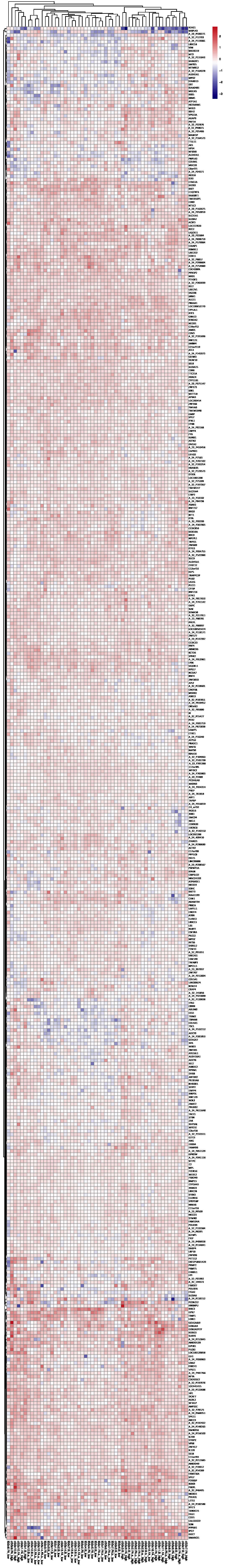

Supplement: S2 Fig — (TIF) [file pone.0199314.s005.tif]

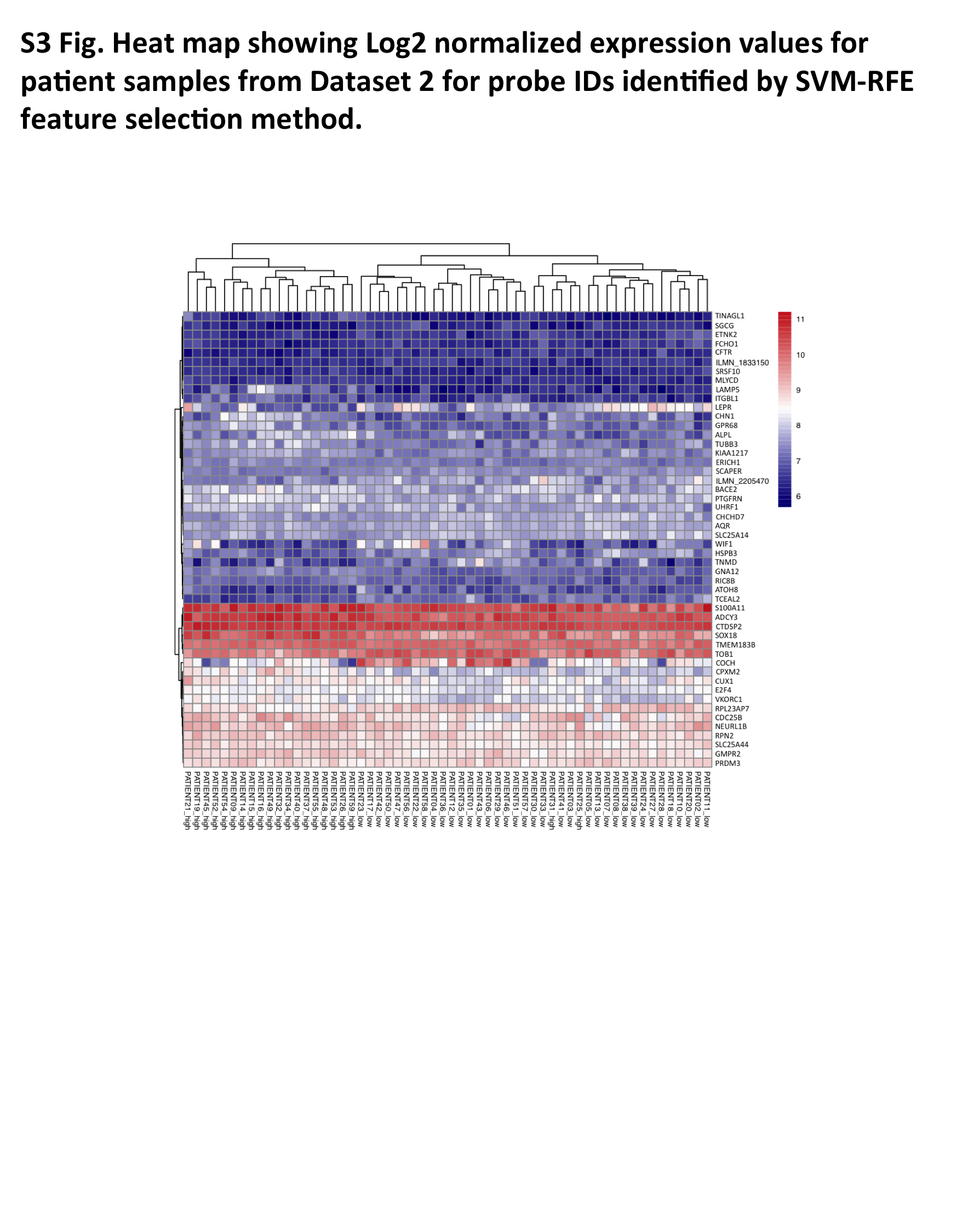

Supplement: S3 Fig — (TIF) [file pone.0199314.s006.tif]
